# scSensitiveGeneDefine: sensitive gene detection in single-cell RNA sequencing data by Shannon entropy

**DOI:** 10.1186/s12859-021-04136-1

**Published:** 2021-04-22

**Authors:** Zechuan Chen, Zeruo Yang, Xiaojun Yuan, Xiaoming Zhang, Pei Hao

**Affiliations:** 1 College of Life Sciences, Shanghai University, Shanghai, China; 2 Key Laboratory of Molecular Virology and Immunology, Institut Pasteur of Shanghai, Chinese Academy of Sciences, Shanghai, China; 3 Natural Medicine Institute of Zhejiang YangShengTang Co., Ltd., No. 181, Geyazhuang, Xihu District, Hangzhou, Zhejiang China

**Keywords:** Sensitive genes, Single-cell RNA sequencing, Stochastic gene expression, Unsupervised clustering

## Abstract

**Background:**

Single-cell RNA sequencing (scRNA-seq) is the most widely used technique to obtain gene expression profiles from complex tissues. Cell subsets and developmental states are often identified via differential gene expression patterns. Most of the single-cell tools utilized highly variable genes to annotate cell subsets and states. However, we have discovered that a group of genes, which sensitively respond to environmental stimuli with high coefficients of variation (CV), might impose overwhelming influences on the cell type annotation.

**Result:**

In this research, we developed a method, based on the CV-rank and Shannon entropy, to identify these noise genes, and termed them as “sensitive genes”. To validate the reliability of our methods, we applied our tools in 11 single-cell data sets from different human tissues. The results showed that most of the sensitive genes were enriched pathways related to cellular stress response. Furthermore, we noticed that the unsupervised result was closer to the ground-truth cell labels, after removing the sensitive genes detected by our tools.

**Conclusion:**

Our study revealed the prevalence of stochastic gene expression patterns in most types of cells, compared the differences among cell marker genes, housekeeping genes (HK genes), and sensitive genes, demonstrated the similarities of functions of sensitive genes in various scRNA-seq data sets, and improved the results of unsupervised clustering towards the ground-truth labels. We hope our method would provide new insights into the reduction of data noise in scRNA-seq data analysis and contribute to the development of better scRNA-seq unsupervised clustering algorithms in the future.

**Supplementary Information:**

The online version contains supplementary material available at 10.1186/s12859-021-04136-1.

## Background

Recent years have seen the rapid spread of scRNA-seq technology [1] in various fields. When compared with traditional RNA-seq (also called bulk RNA-seq), scRNA-seq technology requires fewer samples and allows us to obtain the transcriptome on a single-cell level for more subtle biological differences [2]. In scRNA-seq data, it is challenging to remove technical noise, which is typically confounded with noise originating from the underlying biology of processes at the single-cell level. The technical noise caused by experimental factors, including sequencing time and the states of tissues, are called the batch effect [3]. Except for the batch effect, single-cell expression variability has been increasingly discussed [4]. According to previous studies, the cells of the same type and state will still show a cell-to-cell variability in gene expression, which is considered as cellular heterogeneity or single-cell expression variability [5]. Cellular heterogeneity is the external manifestation of stochastic gene expression [6]. Stochastic gene expression contains two parts: the intrinsic fluctuations, due to the randomness inherent to transcription, and the extrinsic fluctuations, including the cell-to-cell variability driven by stochastic molecular interactions and the noise induced by cell differentiation.

The exploration of stochastic gene expression could be dated back to microarray analysis. In 2009, Pei Hao et al. [7] defined genes that showed different sensitivities in expression in response to various biological conditions as sensitive genes. Pei group found that most of the sensitive genes were related to cellular responses to environmental perturbations, including immune responses and cell–cell signaling. However, in Pei’s study, the concept of sensitive genes was broad and incorporated expression fluctuations from multiple sources (biological sample variation, condition variation, and technical variation, etc.). For single-cell analysis, we are more interested in expression fluctuations within the same cell types and states, and we narrow down the concept of sensitive genes to genes that could represent cellular heterogeneity within the same cell types and states. In fact, studies have already identified cellular heterogeneity in scRNA-seq analysis. For instance, Daniel Osorio et al. [8] identified overlapping HVGs in 3 kinds of cell lines, and those genes were enriched in pathways related to the response to environmental stimuli. While previous studies have demonstrated some potential functions of sensitive genes, a complete method to identify sensitive genes and to evaluate their impact on cell type grouping has not been established.

Cell type grouping is crucial to scRNA-seq analysis, for only the correct classification of cells can explain the true biological differences. The unsupervised clustering based on transcriptome similarity has emerged as one of the most powerful applications in scRNA-seq cell type grouping. The application of feature selection and dimension reduction [9] will reduce noise and speed up the calculation process. Feature selection involves the identification of the most informative genes. Some software, like Seurat [10], scan [11], scLVM [12] and scVEG [13], perform dimension reduction and unsupervised clustering by detecting HVGs according to the CV-rank of each gene in all cells [14]. However, most clustering methods will partition the data regardless of their biological meanings, and they often mistake random noise for true structure [15]. Ideally, we hope that cells of the same cell type are homogeneous during cell annotation in scRNA-seq data analysis. However, in most cases, cells of the same type would have cellular heterogeneity, and such random noises would adversely impact the result of unsupervised clustering.

Thus, in this paper, we proposed a method to identify sensitive genes that represent cellular heterogeneity and explored the impact of these genes on cell type grouping. In this method, we used both the CV-rank and the Shannon index, and only genes qualified for both criteria were defined as the sensitive genes. Furthermore, we also explored the functions of sensitive genes in 11 scRNA-seq data sets covering different human tissues.

## Methods

### Data collection and quality control

To verify the reliability of our method to identify sensitive genes and to explore the function of these genes, we downloaded 10 scRNA-seq data sets, generated by the most widely used 10 × Genomics platform [16], in the Gene Expression Omnibus (GEO) database. In addition, we collected one annotated data set, the Zhang T cells data set sequenced by Smart-seq2 [17], to evaluate our methods’ robustness for cross-platform data sets. In total, we collected 11 scRNA-seq data sets from various human tissues, including peripheral blood mononuclear cells (PBMC) [16, 18], tumor-infiltrating T cells [19], renal tubular cells [20], spermatogonial stem cells [21], lung tissues [22], spleen tissues [22], esophagus mucosa [22], liver tissue [23], and cortical organoids [24].

There were several steps in scRNA-seq data analysis, including quality control (QC), normalization, feature selection, dimension reduction, and clustering. First, we performed QC for each data set. In general, low-quality cells with < 500 genes and > 20% of mitochondrial counts were filtered. Besides, we lowered the QC standards for Zheng PBMC68K and Liao Kidney data sets, and only low-quality cells with less than 200 genes were filtered. We changed our QC strategy accordingly because these two data sets had their median genes per cell less than 1000 and were limited by their sequencing technique at that time. Nevertheless, their sequencing quality could still be guaranteed. The information of all data sets was shown in Table 1.

**Table 1:** Basic information of single-cell data sets in this study after QC

Dataset	N.cells	M.gene	Description	Protocol	N.samples	Annotation	References
PbmcBench PBMC1	8512	1910	PBMC	10X CHROMIUM V3	2	Annotated	[18]
PbmcBench PBMC2	8669	2222	PBMC	10X CHROMIUM V3	2	Unannotated	[18]
Zheng PBMC68K	68,509	525	PBMC	10X CHROMIUM	8	Unannotated	[16]
Zhang T cells	9055	2953	Tumor-infiltrating T cells	Smart-Seq2	14	Annotated	[19]
Liao Kidney	22,052	784	Renal tubular cells	10X CHROMIUM V2	3	Unannotated	[20]
Guo Testis	6466	2575	Spermatogonial stem cells	10X CHROMIUM V2	6	Unannotated	[21]
Hemant Lung	61,431	1335	Frozen lung soft tissues	10X CHROMIUM V2	24	Unannotated	[22]
Hemant Spleen	89,082	1030	Frozen spleen soft tissues	10X CHROMIUM V2	19	Unannotated	[22]
Hemant Esophagus	103,495	2144	Frozen esophagus mucosa	10X CHROMIUM V2	23	Unannotated	[22]
Sonya Liver	8078	1147	Fresh human liver tissue	10X CHROMIUM V2	5	Unannotated	[23]
Cleber Brain	14,940	1628	Cortical organoids	10X CHROMIUM V2	4	Unannotated	[24]

### Data preprocessing and the first-time unsupervised clustering

**Fig. 1:**
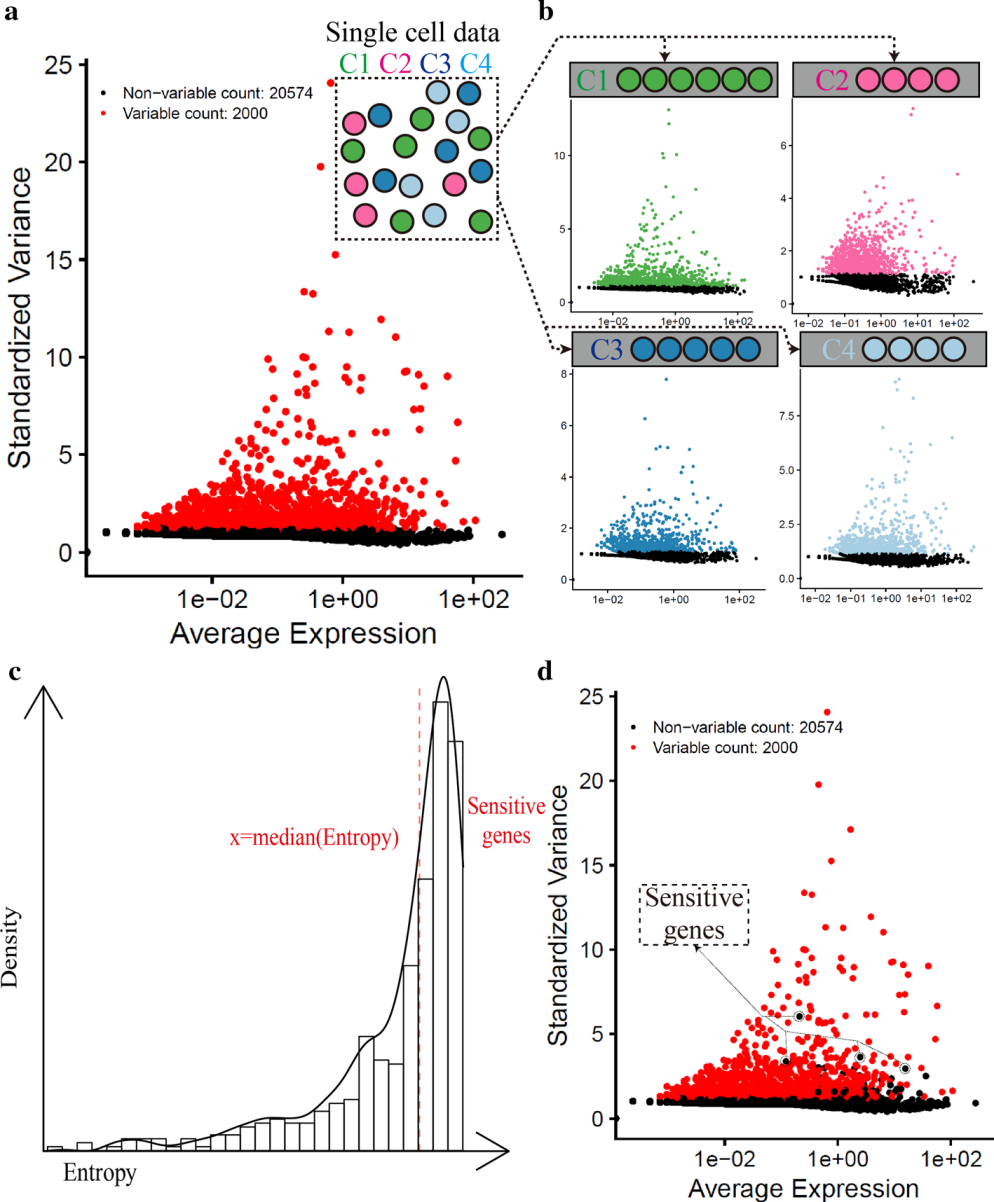
Workflow for sensitive gene identification. **a** After the single-cell sequencing, we obtained expression profiles of various cell types, with different colors representing different cell types. We used Seurat to calculate the CV-rank for all genes in all cells, and the top 2000 genes were defined as HVGs (red); **b** Based on the results of the first-time unsupervised clustering, we detected high CV-rank genes in each cluster; **c** Shannon entropy based on the average expressions of these genes (with high CV-rank in more than half of clusters) among cells in each cluster. The genes with high entropy (higher than the median entropy) were regarded as the sensitive genes; **d** We re-selected the top 2000 HVGs with sensitive genes removed from the expression matrix

After QC, we used Seurat package (Version 3.1.5) in R (Version 3.6.3) to perform the same analysis pipeline for all scRNA-seq data sets. By default, we employed a global-scaling normalization method “LogNormalize” that normalized the feature expression measurements for each cell by the total expression, multiplied this by a scale factor (10,000), and log-transformed the result. Second, to avoid the interference from doublet cells, we identified and removed these doublet cells by using DoubletFinder [25] package (Version 2.0.3) in R. Third, we calculated CV-rank for each gene in all cells and used the top 2000 genes with the highest CV-rank for the downstream analyses, including principal component analysis (PCA) and unsupervised clustering (the Louvain algorithm) [26]. Then, we performed PCA to identify the true dimension of data sets, and we chose as many principal components as possible for the downstream analyses. As for the unsupervised clustering, we chose 0.6 as the default resolution parameter, and this clustering result was defined as the first-time unsupervised clustering result (Fig. 1a–c).

### Sensitive gene identifications

We identified sensitive genes based on the first-time unsupervised clustering result with N clusters. First, we calculated CV for all genes within each cluster and generated a CV-based rank list for each cluster. Second, we retained genes that ranked the top 2000 in more than half of the clusters (≥ N/2) (Fig. 1b). Third, we calculated the average expression values for these genes within each cluster and used these values (each gene has N expression values for N clusters) as the input for the Shannon index calculation (Fig. 1c). The Shannon index formula was given below:

$$\begin{aligned} H\left( x \right) & = - \sum\limits_{i = 1}^{N} {p\left( {x_{i} } \right){\text{log}}p\left( {x_{i} } \right)} \\ p\left( {x_{i} } \right) & = \frac{{x_{i} }}{{\sum\nolimits_{i = 1}^{N} {x_{i} } }} \\ \end{aligned}$$

$N$ represents the cluster number generated from the first-time unsupervised clustering; ${x}_{i}$ is the average expression of a gene for the $i$ th cluster. $p\left({x}_{i}\right)$ is the average expression of the gene in the $i$ th cluster divided by the summation of average expressions of this gene in all clusters. $H\left(x\right)$ is the Shannon index that evaluates the contribution of this gene to cluster-to-cluster differences.

So far, we had generated a Shannon index list for these genes, and we designated median value to be the cut-off point for sensitive-gene selection according to the overall Shannon index distribution. Finally, we designated genes with high CV among half of the clusters and with high entropy (higher than the median entropy) to be the sensitive genes.

### Sensitive gene removal

During feature selection, we removed sensitive genes from the expression matrix and re-selected the top 2000 HVGs with high CV-rank in all cells and redid the unsupervised clustering (Fig. 1d).

### Clustering results evaluation

We utilized two evaluation metrics, the entropy of cluster accuracy (ECA) [27] and the entropy of cluster purity (ECP) [27], to compare clustering results between the first-time unsupervised clustering and clustering after the removal of sensitive genes.

### Enrichment analysis

We performed KEGG [28–30] enrichment analysis for sensitive genes in each data set by using ClusterProfiler [31] package (Version 3.14.3) in R. We gathered statistical results and explored the similarity of sensitive gene distribution in different data sets.

## Results

### Sensitive gene identification and verification

To evaluate the reliability of our method to identify sensitive genes, we analyzed 2 PBMC data sets downloaded from the 10 × Genomics platform, including total 17,181 cells from 4 Human PBMC samples. In the downstream analysis, we combined PbmcBench PBMC1 and PbmcBench PBMC2 data set into the PbmcBench PBMC data set, resulting in a total of 4 samples.

**Fig. 2:**
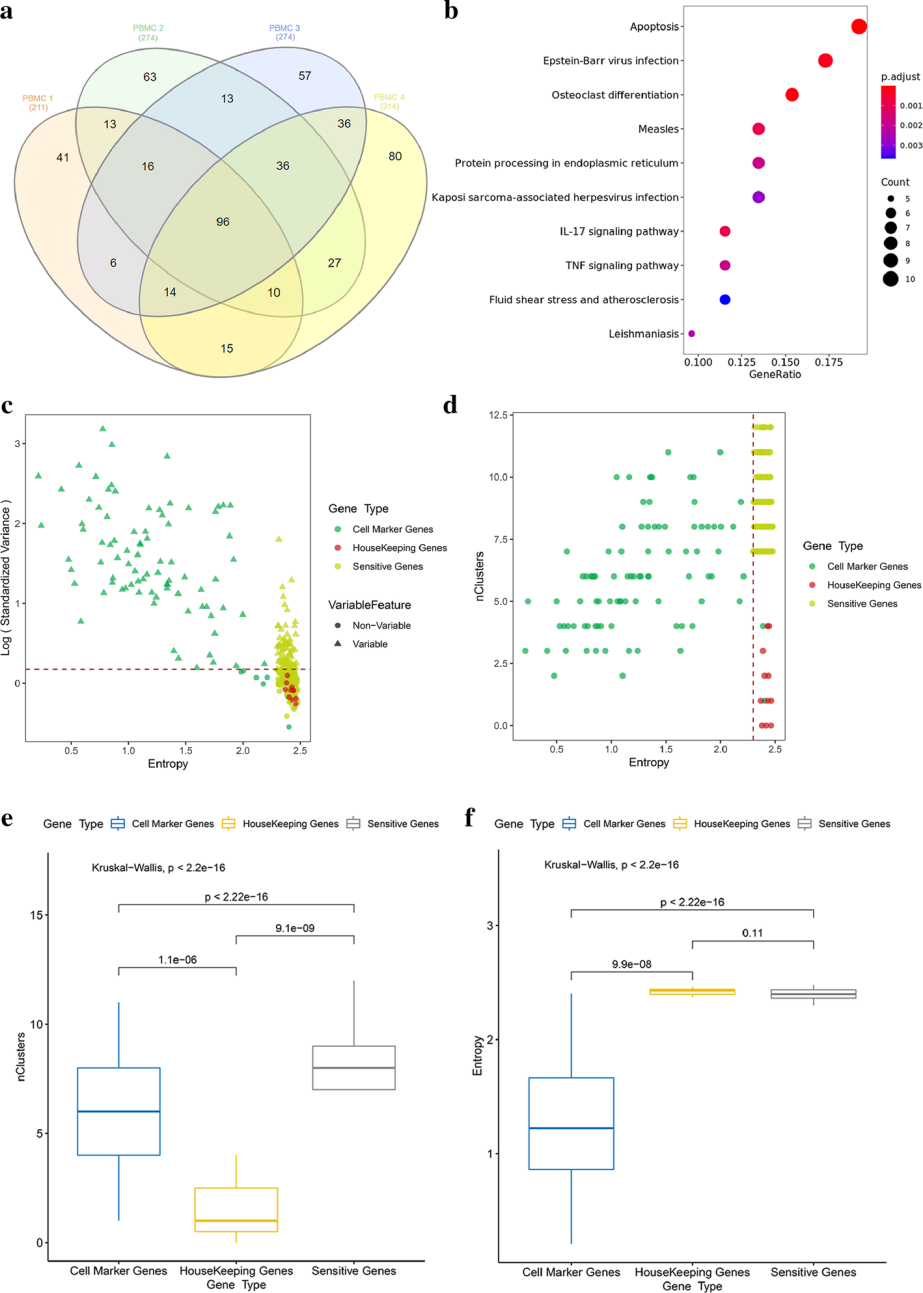
Reliability evaluation of sensitive gene identification in PbmcBench PBMC data set. **a** There were 211, 274, 274 and 314 sensitive genes in each sample of the PbmcBench PBMC data set, with 96 sensitive genes in common; **b** The KEGG enrichment result of these 96 common sensitive genes showed that these sensitive genes were obviously enriched in pathways related to cellular stress response; **c** In the first sample of PbmcBench PBMC1, we compared three types of genes (cell marker genes, HK genes, and sensitive genes) by their entropy and CV-rank in total cells (dotted line represent the threshold of HVGs); **d** We compared these three types of genes by their entropy and number of clusters with high CV-rank (top 2000); **e**, **f** We used the Kruskal–Wallis test to compare these three types of genes from the aspects of entropy and number of clusters with high CV-rank (top 2000)

After the sensitive-gene analysis, we identified 211, 274, 274, and 314 sensitive genes from 4 PBMC samples, respectively. There was a high degree overlap of sensitive genes in these 4 samples, with 96 sensitive genes in common (Fig. 2a). The functional enrichment analysis on these 96 genes showed that the sensitive genes were obviously enriched in pathways related to cellular stress response, such as apoptosis (p.value.adjust = 1.8E−6), epstein-barr virus infection (p.value.adjust = 9.32E−5), antigen processing and presentation (p.value.adjust = 1.2E−3) and fluid shear stress and atherosclerosis (p.value.adjust = 5.1E−3) (Fig. 2b, filtering threshold: p.value.adjust < 0.01).

To better understand the attributes of sensitive genes, we also calculated the Shannon index for two other types of genes, the HK genes and cell marker genes, in the first sample of the PBMC1 data set. For HK genes, we selected 11 HK genes with high expressions (RPKM > 50 in Eli’s paper) identified by Eli Eisenberg [32]; for cell marker genes, we selected the 10 most differentially expressed genes for each cluster in this sample (in total 91 genes, after the removal of duplicated genes) through differentially expressed gene analysis by FindAllMarkers function in Seurat. We compared these three types of genes by considering their entropy and CV-rank in all clusters. We found that: cell marker genes represented the true biological variance, with high CV-rank (the top 2000) in only few clusters and with low entropy; HK genes had constitutive expressions, and thus had low CV-rank in all clusters and with high entropy; Sensitive genes had fluctuated expressions in various types of cells and thus had high CV-rank in more than half of the clusters and with high entropy as shown in Fig. 2c, d. Then, the Kruskal–Wallis test was used to test for significance in their differences in terms of the number of high CV-rank clusters and entropy. We found that sensitive genes had high CV-rank in more clusters compared to cell marker genes (p < 2.22E−16) and HK genes (p = 9.1E−09) (Fig. 2e). As for entropy, there was a significant difference between sensitive genes and cell marker genes, in which cell marker genes had a much lower entropy (p < 9.9E−6). However, there was no significant difference between sensitive genes and HK genes (p = 0.11) (Fig. 2f). Thus, we evaluated the possibility that HK genes would be misidentified as sensitive genes. From Additional file 1: Figure S1, we could see that only the Liao Kidney data set’s samples had a relatively high rate (0.33) of misidentification for these two types of genes, which was likely caused by the fact that this data set only had 3 samples.

### Predict the function of sensitive genes

Even though single-cell sequencing by the 10 × Genomics platform was high-throughput, usually only 500–2000 highly expressed genes per cell could be detected [33]. In general, scRNA-seq data sets from various tissues were quite different, and thus it was difficult to obtain an overlap of sensitive genes from different tissues. Moreover, due to the difference in library preparation, it was also hard to compare sensitive genes across different data sets. Nevertheless, we could easily detect the overlap of sensitive genes in different samples in the same data set.

**Fig. 3:**
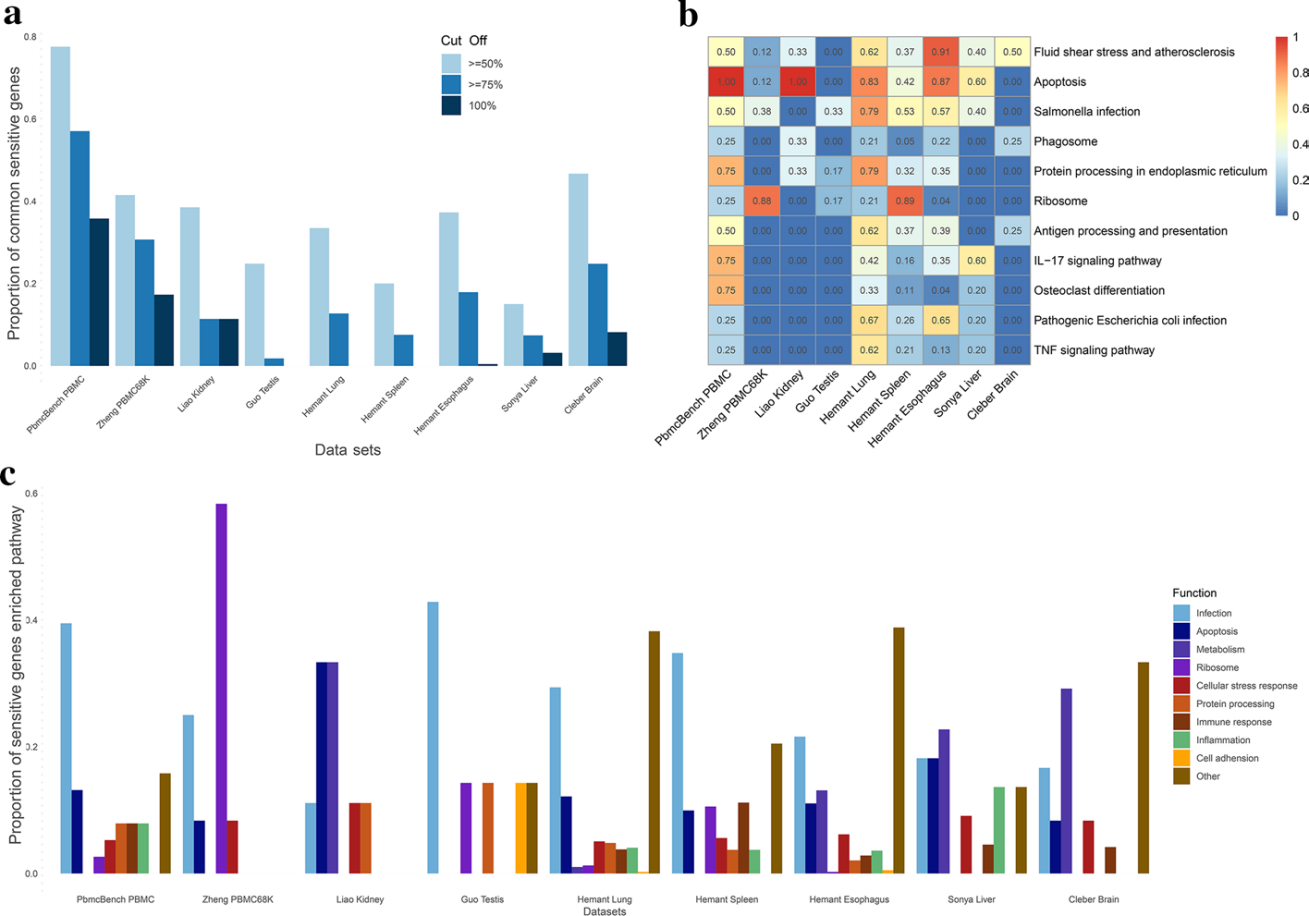
Evaluation and function annotation of sensitive genes in various tissues. **a** The proportion of overlapping sensitive genes (the overlap in greater or equal to 50%, 75% and 100% of samples in a given data set) out of the total number of sensitive genes in all samples (by the union of sensitive genes in all samples in a given data set) for these data sets; **b** The most enriched KEGG signaling pathways (detected in more than half of the data sets) of sensitive genes, with color representing the percentage of samples in each data set with sensitive genes enriched in these signal pathways; **c** We classified these enriched signaling pathways into several main types, including infection, apoptosis, metabolism, ribosome, cellular stress response, immune response, inflammation, protein processing, cell adhesion and other signaling pathways. Most of the sensitive genes were enriched in signaling pathways related to stress response against environmental changes

As shown in Fig. 3a, we calculated the proportion of overlapping sensitive genes (overlap of genes in greater than or equal to 50%, 75% and 100% of samples in a given data set) out of the total number of sensitive genes in all samples (by the union of sensitive genes in all samples in a given data set) for these data sets. From the result, we found that, in most data sets, over 30% of sensitive genes (for this data set) appeared in at least 50% of the samples. Thus, based on the above calculations, we claimed that our method to identify the sensitive genes was robust.

We also explored the functions of sensitive genes of these data sets by conducting the functional enrichment analysis. Although scRNA-seq data sets from various tissues were quite different, the functions of sensitive genes identified from these data sets were nearly consistent. As shown in Fig. 3b, most of the sensitive genes were enriched in pathways related to cellular stress response, including apoptosis, fluid shear stress response, infection, and inflammation response. We further classified these enriched signaling pathways into several main types, including infection, apoptosis, metabolism, ribosome, cellular stress response, immune response, inflammation, protein processing, cell adhesion, and other signaling pathways (Fig. 3c). As expected, most of the sensitive genes, identified from various tissues, were enriched in signaling pathways related to cellular stress response against environmental changes.

### Removal of sensitive genes optimized unsupervised clustering result

To test the influence of sensitive genes on clustering results, we compared the clustering results between the first-time clustering and clustering removing sensitive genes against the ground-truth (cell-label annotations) in two 10 × Genomics scRNA-seq samples of the PbmcBench PBMC1 data set. The PbmcBench PBMC1 data set was stained with a panel of Total-Seq™-B antibodies (BioLegend), which could serve as true cell-type annotations.

**Fig. 4:**
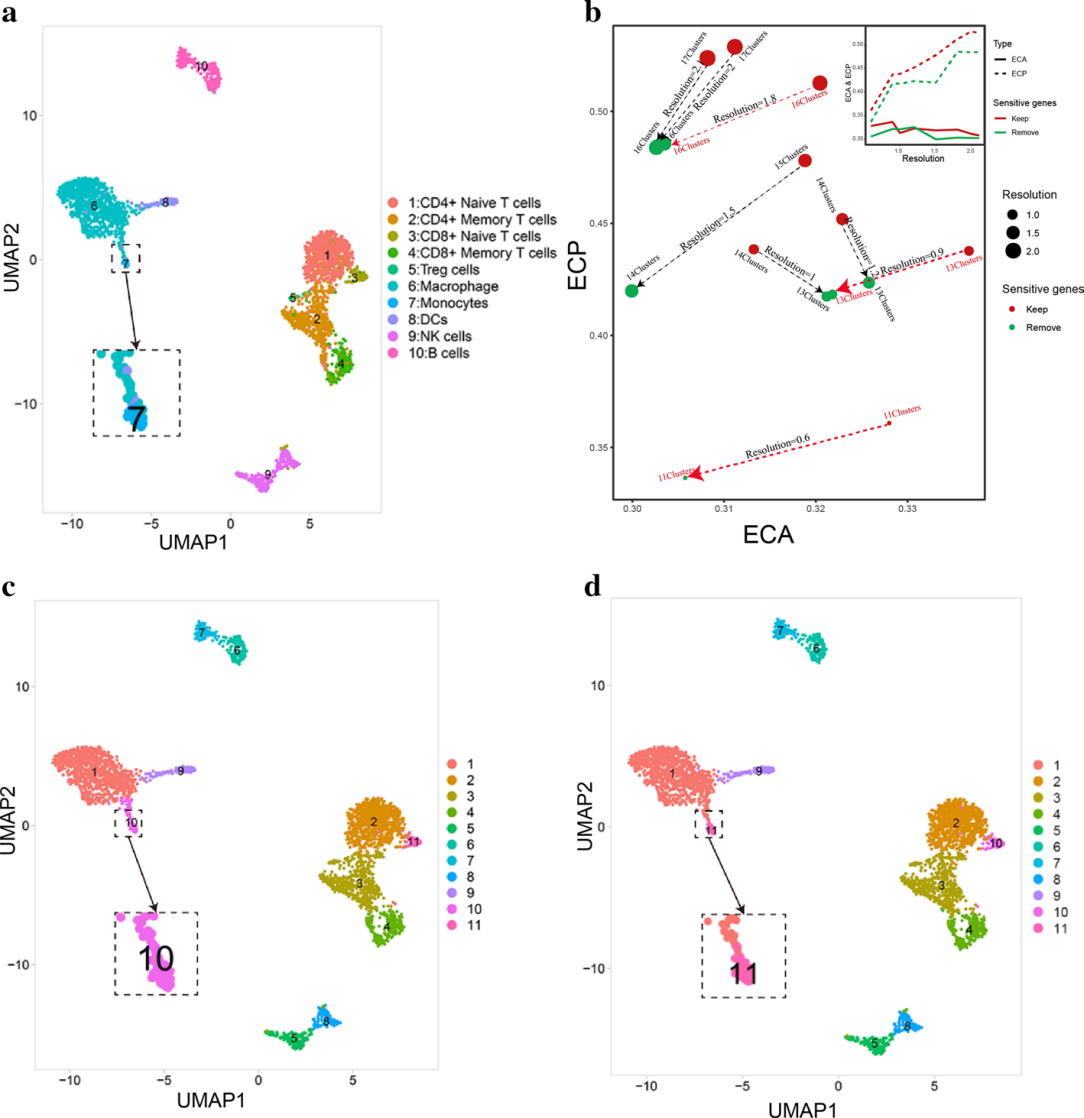
Evaluation of the influence of sensitive genes on unsupervised clustering results. **a** The ground-truth labels with cell-type annotation of the first sample in PbmcBench PBMC1 data set; **b** ECA and ECP values in a series of resolution (0.6, 0.9, 1,1.2, 1.5, 1.8, 2) including and removing sensitive genes. Arrows aimed from the group keeping the sensitive genes to the group discarding the sensitive genes. The paired points in two groups with the same number of clusters were marked by red arrows, the paired points in two groups with different number of clusters were marked by black arrows; **c** The first-time unsupervised clustering result (resolution = 0.6); **d** The unsupervised clustering result (resolution = 0.6) removing sensitive genes

As shown in Fig. 4, there were the ground-truth labels (Fig. 4a), the first-time clustering result (resolution = 0.6) (Fig. 4c), and the clustering result removing sensitive genes (resolution = 0.6) after dimension reduction (Fig. 4d), respectively. We could see that removing sensitive genes enabled us to classify some indistinguishable cell types clearly, such as the monocyte and the macrophage (Fig. 4c, d). Since it was difficult to observe clustering performance directly, we incorporated two evaluation metrics, the ECA and the ECP metrics. While ECA measured the diversity of the ground-truth labels within each cluster assigned by the unsupervised clustering, ECP measured the diversity of clusters within each group from the ground-truth labels. We used both metrics to avoid under-clustering and over-clustering performance, and optimal clustering results would have low values in both ECP and ECA.

Since Seurat could not determine the optimal cluster number, we chose a series of resolutions on the x-axis ranging from 0.6 to 2.1, in the steps of 0.3, and keep the commonly used resolution (0.6, 1 and 2) to control cluster numbers in the unsupervised clustering. Compared with the first-time unsupervised clustering, the result removing sensitive genes had the same number of clusters in these samples (resolution = 0.6, 0.9 and 1.8), which made the results comparable. As shown in Fig. 4b, in the first sample of PbmcBench PBMC1 data set, both metrics showed denoting reductions under resolution 0.6, 0.9, 1.5, 1.8, 2 and 2.1. Under the resolution 1 and 1.2, however, ECA increased whereas ECP decreased. We propose that this might be due to the different numbers of clusters generated under these two resolutions. Under the definition of the ECA measure, more clusters would lead to a lower ECA value. The unsupervised clustering result keeping sensitive genes had one more cluster under resolution 1 and 1.2, and this increase of cluster numbers might overweigh the influence of sensitive genes’ removal on the ECA value and therefore such a condition was not suitable for comparison of ECA and ECP measures. Interestingly, the unsupervised clustering result keeping sensitive genes had one more cluster under resolution 1.5, 2 and 2.1, and both metrics decrease, which mean ECP was hard to compare but ECA did decrease. Thus, we proposed that removing sensitive genes, the result of unsupervised clustering was closer to true cell-type labels, if the clustering results were under the same resolution and of the same number of clusters. Similar results were also observed in the second sample of PbmcBench PBMC1 data set (Additional file 1: Figure S2a–d) and Zhang T cells data set (Additional file 1: Figure S2e–h).

**Fig. 5:**
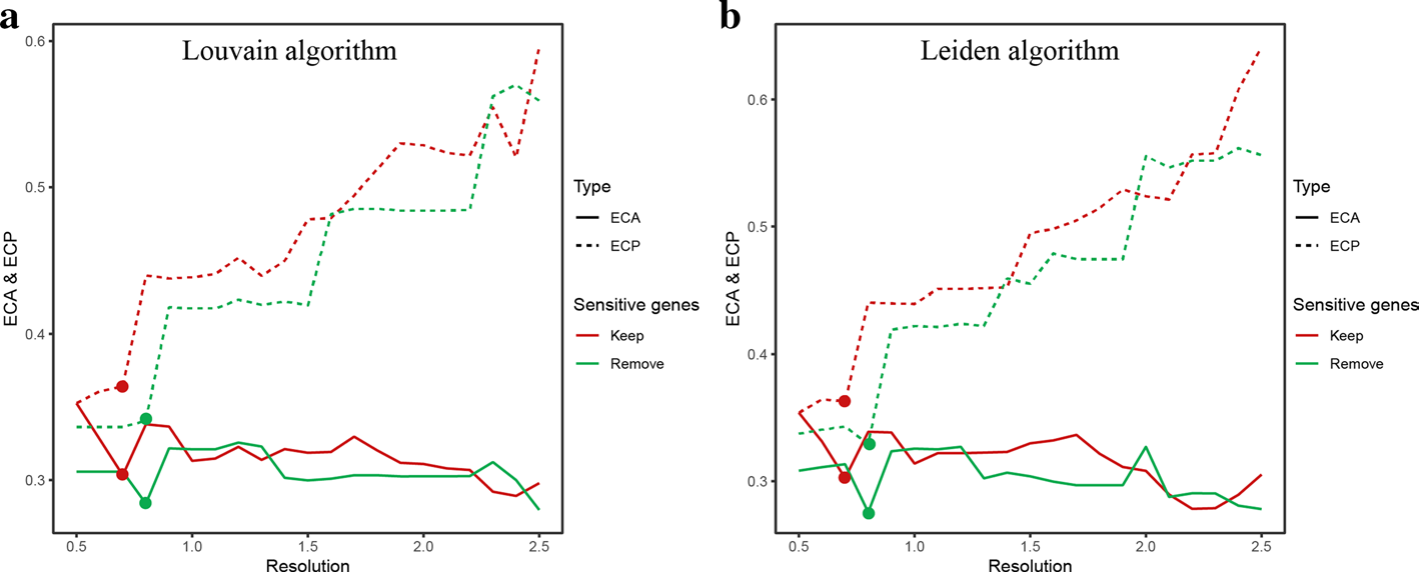
Verification of the influence of sensitive genes with a second clustering algorithm. **a** Using Louvain algorithm for unsupervised clustering to compare ECA and ECP values with a series of resolutions (0.5–2.5, in steps of 0.1) of the first sample in PbmcBench PBMC1 data set; **b** Using Leiden algorithm for unsupervised clustering to compare ECA and ECP values in a series of resolutions (0.5–2.5, in steps of 0.1)

Additionally, we verified our tool with a second clustering algorithm, the Leiden algorithm. We chose a series of resolutions on the x-axis from 0.5–2.5, in the step of 0.1. In both two clustering algorithms, the ECP increased significantly with the increase of resolutions, whereas the curve of ECA was complicated. Interestingly, under resolution 0.8, the clustering result with sensitive genes removed had lower ECA and ECP compared with the total curve (resolution 0.5–2.5) of first-time unsupervised clustering result (Fig. 5a, b).

## Discussion

In summary, we provided a method to identify sensitive genes based on the first-time clustering result. Through the CV-rank within clusters and entropy calculations, we identified sensitive genes with high CV in more than half of the clusters and with high entropy. By applying our methods in various tissues from 10 different single-cell data sets, we found that: 1. there were a large number of overlapping sensitive genes for different samples in the same data set; 2. most of the sensitive genes, though detected from different tissues, were enriched in similar pathways related to cellular stress response. Finally, our study quantified the influence of sensitive genes on the clustering result by using ECA and ECP in three data sets. In general, the result of unsupervised clustering with the sensitive genes removed was closer to true cell-type labels when compared to the first-time clustering.

Our study still had several limitations. First, even though we had improved the clustering results by removing sensitive genes, our result was not completely identical to the ground-truth labels. Besides, limited by the sequencing depth of the 10 × Genomics scRNA-seq, it was hard to identify universal sensitive genes across different tissue samples. In the future, we hope that with the advancement of the single-cell sequencing technique, we would be able to identify cross-tissue sensitive genes and explore their functions.

## Conclusion

The accuracy of the unsupervised clustering result is key to the success of scRNA-seq research. In this paper, we have provided a method to improve the clustering result by identifying and removing sensitive genes. We hope our method will provide new insights into the reduction of data noise in scRNA-seq data analysis and contribute to the development of better scRNA-seq unsupervised clustering algorithms in the future.

## Supplementary Information

**Additional file 1**. Misidentification ratio and tools application in other data sets.

## Data Availability

The scripts and code can be found in https://github.com/Zechuan-Chen/scSensitiveGeneDefine.

## Abbreviations

scRNA-seq: Single-cell RNA sequencing
HVG: Highly variable gene
CV: Coefficients of variation
HK gene: Housekeeping gene
GEO: Gene Expression Omnibus
PBMC: Peripheral blood mononuclear cell
QC: Quality control
PCA: Principal component analysis
ECA: Entropy of cluster accuracy
ECP: Entropy of cluster purity
