# Supplementary material for: scSensitiveGeneDefine: sensitive gene detection in single-cell RNA sequencing data by Shannon entropy
Source: BMC Bioinformatics. 2021 Apr 22;22:211. doi: 10.1186/s12859-021-04136-1 (PMC8063398; doi:10.1186/s12859-021-04136-1)
Supplement: Supplementary file 1 — Additional file 1. Misidentification ratio and tools application in other data sets. [file 12859_2021_4136_MOESM1_ESM.doc]

**Supplementary**

**Figure S1:**

**
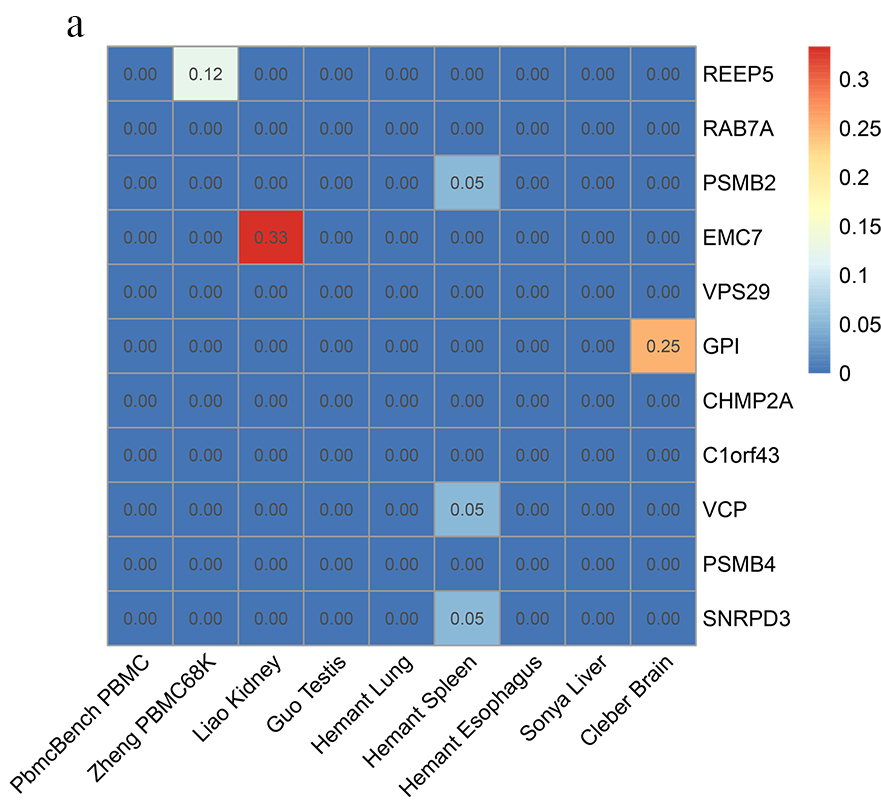
**

a: The heatmap that showed the proportion of samples with misidentification for HK genes and sensitives genes in the total samples for each data set.

**Figure S2:**


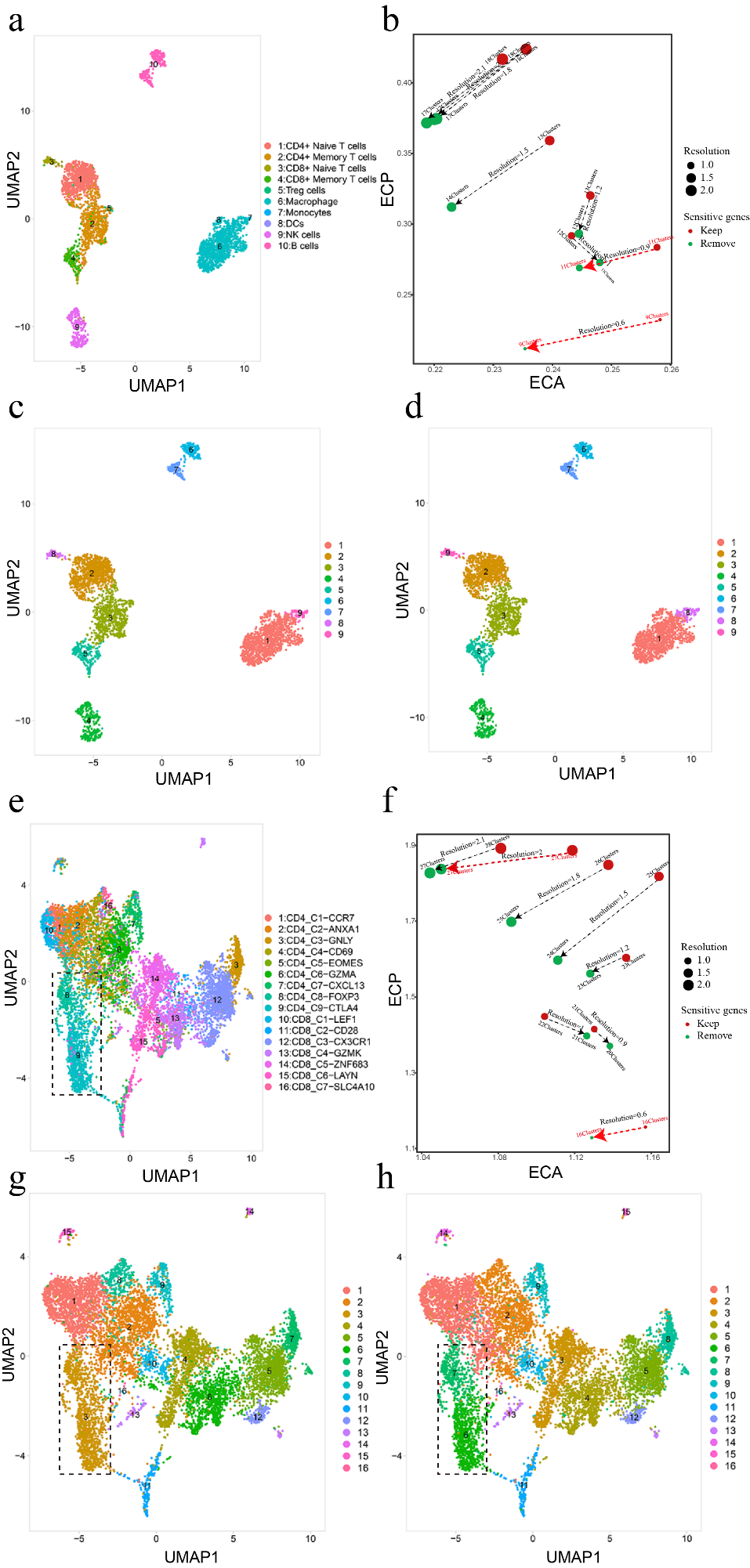


a: The ground-truth labels with cell-type annotation of the second sample in PbmcBench PBMC1 data set; b: Compare ECA and ECP value in a series of resolution. Arrows aimed from the group with keeping sensitive genes to the group with sensitive genes removed. And the paired points in two group had same number of clusters were marked by red arrows; c: The first-time unsupervised clustering (resolution=0.6); d: The unsupervised clustering result (resolution=0.6) after the removal of sensitive genes. e: The ground-truth labels with cell-type annotation of the second sample in PbmcBench PBMC1 data set; f: Compare ECA and ECP value in a series of resolution. Arrows aimed from the group with keeping sensitive genes to the group with sensitive genes removed. And the paired points in two group had same number of clusters were marked by red arrows; g: The first-time unsupervised clustering (resolution=0.6); h: The unsupervised clustering result (resolution=0.6) after the removal of sensitive genes.
